# Q&A: How does jasmonate signaling enable plants to adapt and survive?

**DOI:** 10.1186/s12915-016-0308-8

**Published:** 2016-09-19

**Authors:** Antoine Larrieu, Teva Vernoux

**Affiliations:** Laboratoire Reproduction et Développement des Plantes, University Lyon, ENS de Lyon, UCB Lyon 1, CNRS, INRA, F-69342 Lyon, France

## Abstract

Jasmonates (JAs) are a class of plant hormones that play essential roles in response to tissue wounding. They act on gene expression to slow down growth and to redirect metabolism towards producing defense molecules and repairing damage. These responses are systemic and have dramatic impacts on yields, making JAs a very active research area. JAs interact with many other plant hormones and therefore also have essential functions throughout development, notably during plant reproduction, leaf senescence and in response to many biotic and abiotic stresses.

## 1. What are jasmonates?

**Fig. 1.**
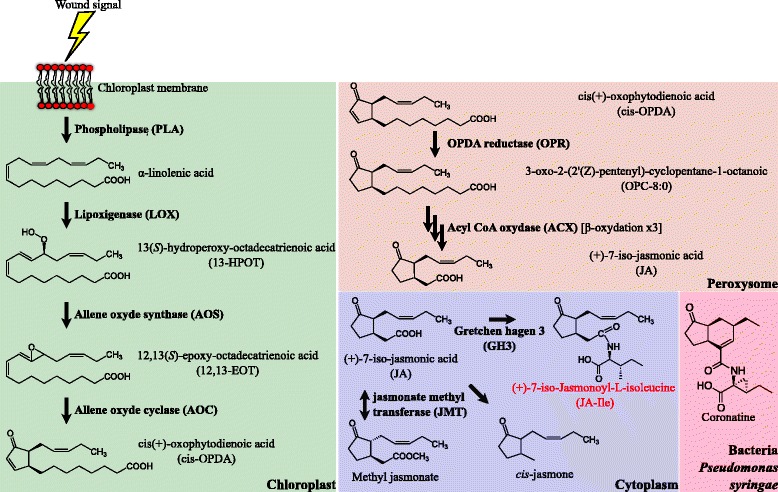
Biosynthesis of JAs and different types of bioactive JAs. Biosynthesis of JAs takes place in three different cellular compartments (chloroplast, peroxisome, and cytoplasm). Refer to Question 4 for further details. Coronatine (*COR*) is not synthesized by plants but by the bacteria *Pseudomonas syringae* (refer to Question 1 for details)

Jasmonates (JAs) are a class of oxidized lipids (oxylipins) that derive from α-linolenic acids (α-LAs). The best-described bioactive JA is (+)-7-iso-Jasmonoyl-L-isoleucine (JA-Ile) [1] (Fig. 1). The biosynthetic pathway that produces JA-Ile (see Question 4, “How are JAs biosynthesized?”; Fig. 1) also leads to the production of several intermediates, such as cis(+)-oxophytodienoic acid (cis-OPDA), as well as secondary metabolites, for instance methyl jasmonate and cis-jasmone, that have important biological functions, some of which are independent of JA-Ile [2–6]. Studies on JAs frequently use coronatine (COR), which is an analogue of JA-Ile, because it can easily be produced by the bacterial pathogen *Pseudomonas syringae* (Fig. 1). COR is a toxin which is used by the pathogen to divert plant defense responses [7, 8], though biotic defense is just one of JAs’ roles.

## 2. When are JAs produced and what are their main functions?

JAs are produced in response to tissue damage, whether caused by necrotrophic pathogens, insects, herbivores, or mechanical stress. Interestingly, in response to a local stimuli (i.e., an insect bite) it is observed that tissues located distally from the bite also produce defense molecules [9]. The systemic effects of JAs are described in more detail in Question 11 (“How does the wound signal spread throughout the plant?”). It has been shown that JA signaling triggers genome-wide changes in gene expression [10] and defects in this pathway reduce plant fitness in the wild (reviewed in [11]). JAs are also produced during several developmental processes and one of the landmark phenotypes of JA insensitive or biosynthetic mutants in several plant species is male (e.g., *Solanum lycopersicum*) or female (e.g., *Arabidopsis thaliana*) sterility, indicating an important function in fertility [12]. However, they were initially of interest due to their odorant properties.

## 3. When were JAs discovered and their role as hormones established?

In 1899 Hesse and Müller purified an unknown ketone from *Jasminum grandiflorum* essential oil extracts and called it jasmone [13]. Its chemical structure was solved in 1933 by Ruzicka and Pfeiffer [14]. However, purified jasmone, combined with other known compounds also isolated from *Jasminum* essential oil, didn’t have the characteristic scent of jasmine flowers, suggesting one or more constituents were missing. In 1962, methyl jasmonate was eventually isolated and its structure determined, and it was shown to be the missing odorant molecule [15]. It took another 20 years before biologists observed that JAs had effects on leaf senescence and seedling growth [16, 17]. The identification of the JA biosynthetic pathway was already almost complete in 1992 [18], while the first *A. thaliana* JA resistant mutant, *coi1* (for CORONATINE INSENSITIVE 1), was isolated in 1994 [19]. The gene was cloned in 1998 [20] and formally identified as the only (thus far) JA receptor 11 years later [21, 22].

## 4. How are JAs biosynthesized?

The JA biosynthetic pathway, also called the octadecanoids pathway (the starting point is the 18 C fatty acid α-linolenic acid 18:3 (α-LA)), takes place in three subcellular compartments: first in the chloroplast, then the peroxisome, and finally the cytoplasm (Fig. 1). The very first step consists in the release of α-LA from galacto- and phospholipids localized at the chloroplast membrane by the action of phospholipases (PLAs), which include DEFECTIVE IN ANTHER DEHISCENCE 1 (DAD1) in *A. thaliana* [23]. Subsequently, the oxidation of the polyunsaturated fatty acids α-LA by 13-LIPOXYGENASE (LOX) leads to 13-hydroperoxy-9,11,15-octadecatrienoicacid (13-HPOT) [24–26]. Two different enzyme families, termed ALLENE OXYDE SYNTHASE (AOS) and ALLENE OXYDE CYCLASE (AOC), successively convert 13-HPOT into the stable cis(+)-oxophytodienoic acid (cis-OPDA) intermediate [27–30]. The next steps of JA biosynthesis take place in the peroxisome. How cis-OPDA is addressed to this subcellular compartment is largely unknown. So far, only one gene, COMATOSE, a peroxisome-localized protein of the ATP binding cassette (ABC) transporter class, has been linked with JA transport to this subcellular compartment [31, 32]. However, as loss of function mutants (in *Arabidopsis*) can still make some JA, there are most likely other transporters involved. In the peroxisome, cis-OPDA is reduced by an OPDA REDUCTASE (OPR) and then undergoes three rounds of β-oxydation by ACYL-CoA OXIDASE (ACX) enzymes leading to the production of jasmonic acid (JA) [33, 34]. JA is then exported though an unknown route to the cytoplasm where it can be modified by several enzymes (reviewed in [2]). The best described belongs to the class of GRETCHEN HAGEN 3 s (GH3s), which conjugates JA with various amino acids but most notably isoleucine, leading to the bioactive JA-Ile molecule [1, 35].

## 5. How is JA-Ile perceived by cells?

**Fig. 2.**
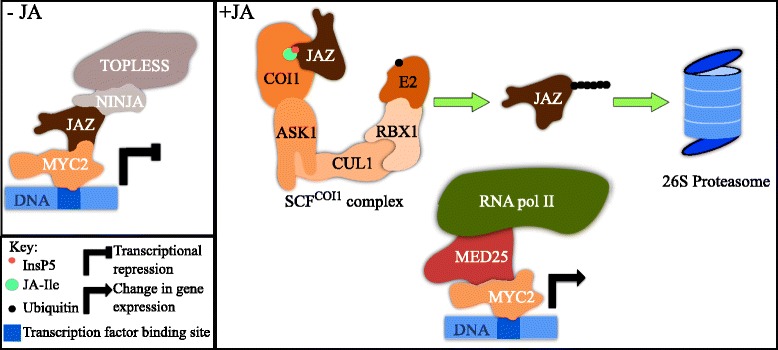
JA signaling. JA perception and signal transduction take place in the nucleus. Refer to Questions 5, 6, and 7 for details. Protein structures are not accurately depicted and structural features are not to scale. For JAZ proteins, the ZIM domain would be on the *top right* portion and the Jas motif on the *bottom right*. Homo/hetero-dimerization of the JAZ proteins is not represented for clarity. The site for JAZ protein polyubiquitination is not known

How plant cells sense JA-Ile has been well documented and is mechanistically very similar to auxin signaling (similarities are reviewed in [36]; Fig. 2). JA-Ile acts as a molecular glue between its co-receptors COI1, an F-BOX E3 LIGASE protein, and JASMONATE ZIM DOMAIN (JAZ) proteins, which act as transcriptional repressors [21, 37, 38]. Most JAZ proteins share two conserved regions: a ZIM domain and a Jas motif [39]. While the ZIM domain mediates protein–protein interactions that regulate JA signal transduction, the Jas motif is involved in JA perception and signal transduction. The crystal structure of a COI1/JA-Ile/JAZ1 complex revealed that high affinity binding of JA-Ile requires a bipartite degron on the JAZ protein [21]. The degron, which encompasses the Jas motif, is a 20-amino acid sequence that is required to interact with COI1. The JAZ degron sequence consists of a conserved α-helix for COI1 docking and a loop region to trap the hormone in its binding pocket. Sheard and colleagues [21] also showed that inositol pentakisphosphate (InsP5) is a key component of the complex as it interacts with both COI1 and the JAZ degron adjacent to the hormone. Interestingly, it was also shown that the affinity of COI1 and JAZ co-receptors is higher for coronatine than JA-Ile. These structural studies were either predicted by molecular modeling [22] or confirmed in further papers [40]. Remarkably, all the structural data allowed for the rational design of novel antagonists of JA signaling [41], preventing the next step in JA signaling.

## 6. What happens when JA-Ile binds its co-receptors?

As for perception, the canonical signaling pathway of JA-Ile is also remarkably similar to auxin [36]. The JA-Ile-dependent interaction between COI1 and JAZ proteins leads to JAZ degradation by the ubiquitin proteasome system (UPS) [37, 38, 42] (Fig. 2). Polyubiquitination of JAZ proteins has been indirectly observed using proteomic approaches [43–45] and their degradation is proteasome dependent as MG132 treatments stabilize them [38, 42]. The ZIM domain of the JAZ proteins notably controls their homo- or hetero-dimerization, which is essential for their function [46, 47]. The ZIM domain also controls the recruitment of TOPLESS (TPL) co-repressors through a protein termed NINJA (NOVEL INTERACTOR OF JAZ) [48]. The Jas motif confers JA sensitivity to JAZ proteins and also controls the interaction with target transcription factors (TFs; reviewed in [49]). Overall, the chain of events from perception to responses at the genomic level is achieved in three distinct steps: 1) JA-Ile is perceived by its co-receptor complex; 2) JAZ proteins are degraded by the proteasome; 3) downstream genes are activated through the release of specific TFs (Fig. 2).

## 7. How is the transcription of JA-Ile response genes regulated?

Amongst the many TFs that interact with JAZ proteins, the basic helix-loop-helix (bHLH) TFs MYC2, MYC3, and MYC4 have been studied extensively [50–52]. When JAZ proteins are stable (in the absence of JAs), genes downstream of promoters bound by MYCs are kept silent because of the recruitment of TPL via NINJA [48] (Fig. 2). Structural studies have revealed that, in this situation, the Jas motif of JAZ9 forms a complete α-helix that triggers a conformational change at the N-terminal side of MYC3. This interaction, which is structurally different than that between JAZs and COI1, inhibits the recruitment of the MED25 subunit of the mediator complex. Upon release of TPL repression after the degradation of JAZ proteins, MYC3 TFs recruit the MEDIATOR complex to trigger transcription of downstream genes by the RNA pol II [53, 54]. JAZ proteins interact with other types of TFs (reviewed in [49]) and it will be interesting to see whether the repression mechanisms are conserved. Altogether, the two mechanisms described, namely epigenetic silencing by TPL and direct repression of RNA pol II recruitment by JAZs, ensure that JA-responsive genes regulated by MYC TFs are kept silent in unstressed conditions [54] (Fig. 2).

## 8. What are the genes downstream of JA signaling and how do they enable plants to adapt and survive?

**Fig. 3:**
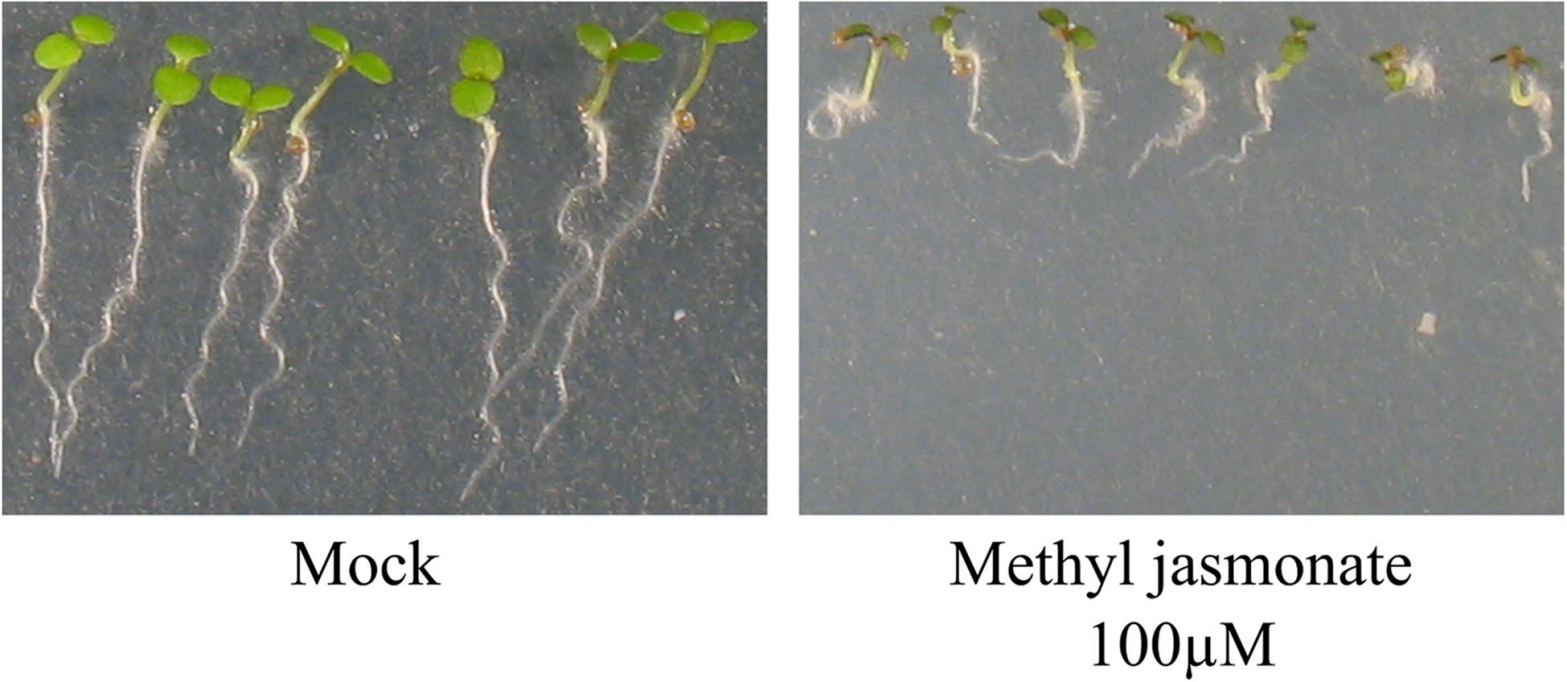
Phenotypes of plants treated with JAs. Plants treated with methyl jasmonate display a dwarf phenotype also called the ‘bonsai effect’. Methyl jasmonate does not seem to be bioactive by itself but is converted to the bioactive JA-Ile *in planta*

The transcriptional changes induced by JAs on their own, for instance in response to methyl jasmonate in cell culture, will primarily affect genes involved in JA signaling (JAZs, MYC2) and in second wave genes involved in metabolism and cell cycle progression [55]. Noir et al. [56] reported that JAs restrain leaf growth by inhibiting G1/S transition, thereby reducing cell number, and by repressing the onset of endoreduplication, which consequently affects cell size. These effects are clearly visible in plants mechanically wounded several times, which display a dwarf phenotype also termed the “bonsai effect” (Fig. 3) [57]. The reduction in growth is not limited to direct effects on cell cycle and cell expansion but is also due to cross-talk between JAs and gibberellins (GAs) [58, 59].

JAs are also important during the responses to pathogens and herbivores. In these situations, wounding is associated with herbivory- or pathogen-associated molecular patterns (H/P AMPS) [60]. These completely change the transcriptomic landscape, allowing plants to respond specifically to various stresses, which increases fitness [10, 61–63].

One of the key effects reported in several studies is the “priming” of JA signaling and, more generally, stress responses [64]. Also called “stress memory” [65, 66], it is the basis of systemic acquired resistance (SAR) in plants [67]. The basic idea is that plants exposed to various types of biotic and abiotic stresses will be able to respond faster and more efficiently to future stresses (recent reports include [68, 69]). Important classes of molecules that prime crop defenses (elicitors) include herbivore-induced plant volatiles [70, 71] but also inorganic elements such as silicon [72]. These notably require JA signaling and allow plants to adapt and survive in a challenging environment.

## 9. Do JAs signal through pathways other than the canonical COI1-JAZ?

Several reports clearly suggest JAs might signal through at least one other pathway that is independent of COI1 and that involves the biosynthetic intermediate OPDA (reviewed in [2]). For instance, transcriptomic studies have revealed that several genes are regulated by OPDA, but in a COI1-independent manner [4]. These notably provide a mechanistic explanation for the observation that OPDA but not JA-Ile is involved in basal defense of tomato plants against *Botrytis* [73]. In terms of evolution, it has been shown that the moss *Physcomitrella patens*, which does not produce JA-Ile but accumulates OPDA, has reduced fertility in *aoc* mutants, a phenotype that is associated with JAs in higher plants [74]. Besides OPDA, other JA metabolites have COI1-independent roles, for instance, cis jasmone [3] and O glucosylated forms of JA [75]. More generally, other classes of oxylipins, which are not JAs but also derive from α-LA, participate in defense responses but also regulate developmental processes such as root growth and root branching [76]. In the near future, the identification of a receptor/receptors or signaling components will be necessary to provide a molecular basis for these observations.

## 10. How is signaling via JAs switched off?

Inactivation of JA signaling takes place at least at two different levels. First, in response to JA treatments or wounding, JAZ repressors are amongst the first genes to be induced [37]. This induction provides a negative feedback loop that aims at switching off JA responses rapidly. Second, inactivation of the bioactive JA-Ile can be achieved either by hydroxylation (12-OH-JA-Ile) and/or carboxylation (12-COOH-JA-Ile) of JA-Ile [77–79] or by the hydrolysis of Ile from JA [80, 81]. The catabolism of JA-Ile in 12-OH-JA-Ile and 12-COOH-JA-Ile occurs relatively fast, as high amounts of these two metabolites can be measured 90 min after wounding a leaf in *Arabidopsis* [82]. Interestingly, the genes involved in inactivation of JA signaling, CYP94B1, B3, and C1, are themselves induced by wounding and jasmonates [83]. The hydrolysis of JA-Ile was shown to be mediated by two amido hydrolases in *Arabidopsis*, ILL6 and IAR3, and by an IAR3 homologous gene in *Nicotiana attenuata*, NaJIH1 [80, 81]. Strikingly, plants silenced for NaJIH1 had increased resistance to insects, clearly suggesting an important biological function for this protein in protection.

## 11. How does the wound signal spread throughout the plant?

The first report of systemic wound responses in plants was in 1972 from the laboratory of Clarence Ryan [9]. The authors demonstrated that beetle feeding or mechanical wounding of tomato or potato plants triggers the accumulation of high amounts of protease inhibitors (PIs) in not only wounded but also unwounded leaves. It was proposed that PIs function to repel insects from eating the plant and were therefore a defense mechanism. Twenty years later, JAs were shown to be the inductive signal triggering the production of PIs [6, 84]. However, how the wound signal is triggered and how fast it is transported to different parts of a plant in the absence of a nervous system only began to be understood much later.

The actual dynamics of the phenomenon were described using very sensitive detection methods, which showed that the spread of the wound response signal moves at a speed of 3.4–4.5 cm.min^−1^ as measured by the production of JA distally to the wounding site (~0.1 cm.s^−1^) [85]. This is two orders of magnitude slower than the conduction velocity of neurons involved in nociception in humans (~3–30 m.s^−1^) [86]. There is evidence that JAs can be transported through vascular tissues in response to wounding [87], which is referred to as the cell non-autonomous pathway [88]. Measurements made using mutants and radiolabeled JA-Ile showed that this is unlikely to involve transport of JA-Ile or OPDA. The cell-autonomous pathway, on the other hand, implies that a signal which is not JA triggers JA production distally, for instance, via electrical signals [89] or hydraulic movements [90]. In 2013 Mousavi and colleagues showed that GLUTAMATE LIKE RECEPTOR genes (GLRs) are essential for the systemic response in *Arabidopsis* [91]. The authors showed that wound activated surface potentials (WASPs) were detected in leaf-to-leaf wound signal transmission and that these were essential to trigger wound responses in distal leaves. The speed at which the signal is transmitted fits with previous observations (5 cm.min^−1^).

More recently, using a fluorescent biosensor to monitor JA responses after wounding, it was shown that systemic signaling from shoots to roots after wounding takes places in two successive waves [42]. The first wave is rapid (<5 min) and triggers a slight degradation of JAZ proteins. The second wave is slower (>30 min) and triggers a much stronger reduction in JAZ proteins. In this experimental set-up, the velocity at which the first wave is transmitted is in a similar range as previously described (≥1 cm/min). Other studies using an output reporter of JA signaling (the promoter of JAZ10) have shown that long distance shoot to root wound signaling is transmitted through the vasculature and then is radially transmitted to the outer tissues [52, 92]. Importantly, the question of whether WASPS are the only systemic signal remains open. Nevertheless, it is clear that these systemic responses are essential to allow plants to survive in the wild, notably by triggering the production of defense molecules and by priming defense responses [9].

## 12. To what extent do JAs affect crop yields and how can we reduce their effects?

Overall, estimates on the proportion of crop losses due to biotic and abiotic stresses range from 20 to 40 % of global yields [93, 94]. Within their natural environments, crops are challenged by a combination of stresses that occur simultaneously or sequentially [62]. In order to understand better how crops cope with such stresses and to find solutions to reduce crop losses, there is a clear need for more integrative analyses that combine multiple stresses (most recent examples include [63, 64]). In addition, repeated wounding has been shown to be required in order to impact root growth in *Arabidopsis* seedlings [52]. Repetitive stresses are frequent and studies looking at the impact of these in relation to the developmental stages of the plants are needed.

## 13. What are the main avenues of research on JAs?

Our understanding of JA biosynthesis, perception, signaling, and catabolism have progressed considerably over the past 10 years. There are now plenty of transcriptomics datasets from different plant species in response to various stresses available online. From these, several genes have been identified that could potentially be interesting for plant breeders as they might enhance resistance to pathogens and resilience to unfavorable weather. Fundamental research now needs to focus on understanding how plants integrate various types of stresses and how they prioritize their responses as in a real life situation crops will typically not face a single, isolated stress but a combination of stresses that might occur sequentially or at the same time. Publications in this area are more and more frequent and the next few years should see these numbers grow even more.

Also, despite the clear evidence that other pathways exist in parallel to the canonical COI1-JAZ module, there are still no molecular mechanisms to explain how these work *in planta*. Transcriptomic studies, as well as evo/devo analyses of JAs signaling in plants, might give a hint, especially since the JA biosynthetic pathway appeared progressively during evolution. Importantly, the identification of stress-specific signaling pathways will allow for the development of biosensors that will help plant growers to monitor their crops more precisely and provide accurate information for when treatments are required to reduce losses.

Finally, further research is required on the transmission of the systemic signal. Are there GLR genes in other plant species that fulfill the same function? Can these be used by crop breeders to select for crops less susceptible to wounding and other stresses? It will be essential to dissect these research areas further for the agriculture of tomorrow.

## 14. Where can I found out more?

There are many reviews that cover all the aspects of JAs, from biosynthesis, signaling, and catabolism to biological function. The most recent contributions include [2, 45, 95, 96]. The review by Claus Wasternack and Bettina Hause [12] and the more general review on the roles of plant hormones in defense responses [67] are the most cited papers in the field of JAs with over 70 citations per year (Web Of Science, search for “jasmonates” as topic, refined to “reviews”).
